# Gels in Medicine and Surgery: Current Trends and Future Perspectives

**DOI:** 10.3390/gels6040048

**Published:** 2020-12-03

**Authors:** Luca Fiorillo, Giovanni Luca Romano

**Affiliations:** 1 Department of Biomedical and Dental Sciences, Morphological and Functional Images, School of Dentistry, University of Messina, Policlinico G. Martino, Via Consolare Valeria, 98100 Messina, Italy; 2 Multidisciplinary Department of Medical-Surgical and Odontostomatological Specialties, University of Campania “Luigi Vanvitelli”, 80121 Naples, Italy; 3 Department of Biomedical and Biotechnological Sciences (BIOMETEC), Section of Pharmacology, University of Catania, 95124 Catania, Italy; giovanniluca.romano@unict.it

Gel is a two-phase elastic colloidal material, consisting of a dispersed liquid incorporated in the solid phase. The liquid “lives” in the structure constituted by the solid, which in turn exploits the surface tension of the liquid in order not to collapse. Gels are prepared by cooling a colloidal solution or by rapid reaction with a high concentration of reagents in the liquid phase [1]. Gels in medicine and surgery have been used for a long time, both for the production of medicaments with different pharmacological purposes, and to have substitutes, biomaterials, capable of promoting regeneration or improving the aesthetic performance of a tissue [2].

Gels are currently present in the form of different formulations, the latter, unlike liquid substances or ointments, have the main advantage of remaining stable on the area to be treated and resisting evaporation for longer, so as to achieve their effect; for example, some gel formulation could be used in mucosae thanks to their mucoadhesive properties. There are gel formulations useful as antimicrobials [3,4,5,6], others as anti-inflammatories or still others as anesthetics. Some formulations have also been proposed to promote wound healing or to promote hemostasis following skin lesions [7].

The recent Sars-CoV-2 pandemic has also led to a very high demand for disinfectant gels. A hand sanitizer is a liquid, gel, or foam used to reduce infectious agents on the hands. There are different formulations, which can be alcohol-based or alcohol-free. The alcohol-based version is included in the World Health Organization’s (WHO) Essential Medicines List, which is the list of WHO deemed safest and most effective medicines in a healthcare system. Compared to washing hands with soap and water, the use of the disinfectant has several advantages and disadvantages associated with both the different sanitation method and the different chemical composition of the products in the two cases, so that each method lends itself to different uses [8,9]. Alcohol-based versions typically contain a combination of isopropyl alcohol, ethanol (ethyl alcohol), or n-propanol. Most products contain between 60% and 80% alcohol. The versions containing from 60% to 95% alcohol by volume are indicated as the most effective from an antiseptic point of view, but they are flammable. In addition to alcohol (ethanol, isopropanol or n-propanol), hand sanitizers may also contain ingredients such as:

- Additional antiseptics such as chlorhexidine and quaternary ammonium derivatives;
- Sporicidal such as hydrogen peroxides which eliminate bacterial spores that may be present in the ingredients;
- Emollients and gelling agents (such as glycerol) to reduce skin dryness and irritation;
- A small amount of sterile or distilled water;
- Sometimes foaming agents, dyes or perfumes, which are generally not recommended due to the risk of allergic reactions;
- Alcohol-free versions typically contain povidone iodine, benzalkonium chloride or triclosan, and are believed to be less effective than alcohol-based ones [10,11].

Hyaluronic acid gel injections are used together with collagen protein injections in surgery and cosmetic dermatology to eliminate wrinkles and prevent skin aging. In otological surgery, hyaluronic acid is used as a regenerator of perforated tympanic membranes, in ophthalmic surgery for the production of artificial tears and operations on the vitreous body of the eye, in arthrology as an anti-inflammatory lubricant and preservative of the synovial fluid of the joints [12,13]. An alternative is represented by the agarose gel. As for its composition, agarose is a gel made up of a mix of sugars and proteins. The molecule is extracted from agar-agar, a gelling agent of natural origin derived from red algae, particularly widespread in Japan. The advantage of the latter, more recent gel is due to the fact that a very limited quantity of it is used and an ultra-thin needle is used: if hyaluronic acid is injected with a one-milliliter syringe, for agarose a 0.7 is used. This gel acts as a mousse that expands under the dermis, forming a sort of 3D network capable of retaining both molecules and liquids [14].

In regenerative surgery, on the other hand, blood products gels can be used. The platelet gel, derived from the concentration of plasma, allows greater tissue regeneration and is applicable to many areas of medicine: in orthopedic, ophthalmic, in the treatment of skin ulcers and, of course, in cosmetic surgery. It falls within the scope of bio-stimulation, being the plasma extracted from the patient himself capable of activating regeneration [15,16]. This bio-stimulation produces a significant increase in fibroblasts. They, after being activated, give a higher quality to the skin. In addition, this platelet gel, when injected into the dermis, acts as a catalyst for growth factors, helping to release them later. Among them we remember: PDGF, TGF, IGF I and II, EGF and FGFB [17,18,19,20,21].

Recently, thanks to the digitalization of medicine, and the improvement of 3D printing systems, these have promoted the formulation of new methods [22,23]. The same principle of “printing” has been converted to be able to generate human tissues in the laboratory. This particular application from 3D printing in the biomedical field is called 3D bioprinting. Although still under development, it is assumed that in the future 3D bioprinting could represent the new frontier of tissue and organ transplantation. The tissues “printed” in the laboratory, could in fact be subsequently transplanted by surgery in patients who need a new tissue. A photosensitive gel has been developed that can solidify when exposed to an infrared light ray capable of crossing the body’s tissues without damaging them. Thanks to the three-dimensional control of the laser, it is possible to create/print solid objects inside the body of a living organism. Gel in liquid form can be injected into the tissues of a live animal and solidified in defined structures from outside the body by exposure to a light emitted by a laser that is able to pass through the tissues without damaging them [24].

Surely in recent years the field of medicine and pharmacology are making considerable progress, gel formulations are increasingly used thanks to their properties.

## References

[1] Díaz D.D. (2015). Welcome to Gels—An Interdisciplinary Open Access Journal for a Growing Scientific Community. Gels.

[2] Fiorillo L., Laino L., De Stefano R., D’Amico C., Bocchieri S., Amoroso G., Isola G., Cervino G. (2019). Dental Whitening Gels: Strengths and Weaknesses of an Increasingly Used Method. Gels.

[3] Meto A., Colombari B., Sala A., Pericolini E., Meto A., Peppoloni S., Blasi E. (2019). Antimicrobial and antibiofilm efficacy of a copper/calcium hydroxide-based endodontic paste against *Staphylococcus aureus*, *Pseudomonas aeruginosa* and *Candida albicans*. Dent. Mater. J..

[4] Meto A., Colombari B., Castagnoli A., Sarti M., Denti L., Blasi E. (2019). Efficacy of a Copper–Calcium–Hydroxide Solution in Reducing Microbial Plaque on Orthodontic Clear Aligners: A Case Report. Eur. J. Dent..

[5] Zuckerman S.T., Rivera-Delgado E., Haley R.M., Korley J.N., von Recum H.A. (2020). Elucidating the Structure-Function Relationship of Solvent and Cross-Linker on Affinity-Based Release from Cyclodextrin Hydrogels. Gels.

[6] Albadr A.A., Coulter S.M., Porter S.L., Thakur R.R.S., Laverty G. (2018). Ultrashort Self-Assembling Peptide Hydrogel for the Treatment of Fungal Infections. Gels.

[7] Dobashi T., Yamamoto T. (2018). Analysis of Heterogeneous Gelation Dynamics and Their Application to Blood Coagulation. Gels.

[8] Fiorillo L., Cervino G., Matarese M., D’Amico C., Surace G., Paduano V., Fiorillo M.T., Moschella A., La Bruna A., Romano G.L. (2020). COVID-19 Surface Persistence: A Recent Data Summary and Its Importance for Medical and Dental Settings. Int. J. Environ. Res. Public Heal..

[9] Cervino G., Fiorillo L., Surace G., Paduano V., Fiorillo M.T., De Stefano R., Laudicella R., Baldari S., Gaeta M., Cicciù M. (2020). SARS-CoV-2 Persistence: Data Summary up to Q2 2020. Data.

[10] Rabenau H., Kampf G., Cinatl J., Doerr H. (2005). Efficacy of various disinfectants against SARS coronavirus. J. Hosp. Infect..

[11] Kramer A., Rudolph P., Kampf G., Pittet D. (2002). Limited efficacy of alcohol-based hand gels. Lancet.

[12] Weis M., Shan J., Kuhlmann M., Jungst T., Tessmar J., Groll J. (2018). Evaluation of Hydrogels Based on Oxidized Hyaluronic Acid for Bioprinting. Gels.

[13] Fiorillo L., Musumeci G. (2020). TMJ Dysfunction and Systemic Correlation. J. Funct. Morphol. Kinesiol..

[14] Ichanti H., Sladic S., Kalies S., Haverich A., Andrée B., Hilfiker A. (2020). Characterization of Tissue Engineered Endothelial Cell Networks in Composite Collagen-Agarose Hydrogels. Gels.

[15] Nastro E., Musolino C., Allegra A., Oteri G., Cicciù M., Alonci A., Quartarone E., Alati C., De Ponte F.S. (2006). Bisphosphonate-Associated Osteonecrosis of the Jaw in Patients with Multiple Myeloma and Breast Cancer. Acta Haematol..

[16] Stacchi C., Lombardi T., Cusimano P., Berton F., Lauritano F., Cervino G., Di Lenarda R., Cicciù M. (2017). Bone Scrapers Versus Piezoelectric Surgery in the Lateral Antrostomy for Sinus Floor Elevation. J. Craniofacial Surg..

[17] Patini R., Gallenzi P., Spagnuolo G., Cordaro M., Cantiani M., Amalfitano A., Arcovito A., Callà C., Mingrone G., Nocca G. (2017). Correlation Between Metabolic Syndrome, Periodontitis and Reactive Oxygen Species Production. A Pilot Study. Open Dent. J..

[18] Liccardo D., Cannavo A., Spagnuolo G., Ferrara N., Cittadini A., Rengo C., Rengo G. (2019). Periodontal Disease: A Risk Factor for Diabetes and Cardiovascular Disease. Int. J. Mol. Sci..

[19] Krifka S., Petzel C., Bolay C., Hiller K.A., Spagnuolo G., Schmalz G., Schweikl H. (2011). Activation of stress-regulated transcription factors by triethylene glycol dimethacrylate monomer. Biomaterials.

[20] Cicciù M., Herford A., Stoffella E., Cervino G., Cicciù D. (2012). Protein-Signaled Guided Bone Regeneration Using Titanium Mesh and Rh-BMP2 in Oral Surgery: A Case Report Involving Left Mandibular Reconstruction after Tumor Resection. Open Dent. J..

[21] Herford A.S., Lü M., Akin L., Cicciù M. (2012). Evaluation of a porcine matrix with and without platelet-derived growth factor for bone graft coverage in pigs. Int. J. Oral Maxillofac. Implant..

[22] Fiorillo L., D’Amico C., Turkina A.Y., Nicita F., Amoroso G., Risitano G. (2020). Endo and Exoskeleton: New Technologies on Composite Materials. Prosthesis.

[23] Fiorillo L., Leanza T. (2020). Worldwide 3D Printers against the New Coronavirus. Prosthesis.

[24] Urciuolo A., Poli I., Brandolino L., Raffa P., Scattolini V., Laterza C., Giobbe G.G., Zambaiti E., Selmin G., Magnussen M. (2020). Intravital three-dimensional bioprinting. Nat. Biomed. Eng..

